# Prevalence of HIV and Viral Hepatitis Markers among Healthcare Workers in the Republic of Guinea

**DOI:** 10.3390/diagnostics13030378

**Published:** 2023-01-19

**Authors:** Yulia V. Ostankova, Alexander N. Shchemelev, Sanaba Boumbaly, Thierno A. L. Balde, Elena B. Zueva, Diana E. Valutite, Elena N. Serikova, Vladimir S. Davydenko, Vsevolod V. Skvoroda, Daria A. Vasileva, Alexander V. Semenov, Elena V. Esaulenko, Areg A. Totolian

**Affiliations:** 1 Saint Petersburg Pasteur Institut of the Federal Service for Surveillance of Consumer Rights Protection and Human Welfare (Rospotrebnadzor), 197101 Saint Petersburg, Russia; 2 Institute of Applied Biological Research of Guinea (IRBAG), Kindia 100 BP 75, Guinea; 3 Centre International de Recherche sur les Infections Tropicales en Guinée, Nzerekore 400 BP, Guinea; 4 Ekaterinburg Research Institute of Viral Infections, State Research Center of Virology and Biotechnology Vector of the Federal Service for Surveillance of Consumer Rights Protection and Human Welfare (Rospotrebnadzor), 620030 Ekaterinburg, Russia

**Keywords:** HIV, viral hepatitis, healthcare workers

## Abstract

Healthcare workers are much more likely to be infected with HIV and hepatitis viruses compared to the general population. Although healthcare workers are more aware of HIV and hepatitis viruses, several countries in Africa lack a comprehensive grasp of disease routes and transmission risks. The aim of this study was to assess the prevalence of the serological and molecular biological markers of HIV and viral hepatitis among healthcare workers in the Republic of Guinea. The study material was 74 blood serum samples collected from healthcare workers who received additional training at the Institute of Applied Biological Research of Guinea (IRBAG, Kindia, Republic of Guinea). The markers examined included HBsAg, HBeAg, anti-HBs IgG, anti-HBcore IgG, anti-HCV qualitative determination, anti-HEV IgM and IgG, anti-HAV IgM and IgG, and anti-HIV. For viral DNA and RNA detection, nucleic acids were extracted from blood serum, and viral presence was inferred using real-time PCR with hybridization fluorescence detection. A high prevalence of viral hepatitis B markers was shown, and significantly fewer cases of viral hepatitis C and HIV were detected. Almost all examined medical workers had anti-HAV IgG antibodies, but no antibodies to hepatitis E virus. Apparently, the identified markers depend on the general prevalence of certain pathogens in the region and are associated with the traditions and characteristics of the country’s residents.

## 1. Introduction

Healthcare workers (HCWs) are at the forefront of the fight for human lives and are highly valued by the population. They are considered a high-risk group for viral infection due to occupational exposure. Occupational exposure can occur through non-intact skin exposure, mucous membrane exposure, or percutaneous injury. The latter is the most common cause (from 66 to 95%) of infection with bloodborne pathogens [1]. About 2.5% of human immunodeficiency virus (HIV) infections and 40% of hepatitis viral groups (HBV, HCV) in HCWs are due to percutaneous injuries.

There is a global shortage of medical workers, especially in low-income countries [2]. The number of qualified HCWs in relation to the patient population, their competence, and their motivation are important factors in the healthcare system [3]. HCWs represent less than 3% of the population in the large majority of countries and less than 2% in almost all low-income countries [4]. The shortage of HCWs in low-income countries may in turn lead to staff having to work longer hours. Longer hours can bring additional cash income for HCWs. However, fatigue can increase, which may result in overworked staff becoming less alert. This, in turn, leads to a greater likelihood of contact with infected blood or other body fluids [1]. It should be noted that infected HCWs who do not follow preventive measures or infection safety guidelines can become a source of infection for patients through unsafe medical injection practices or use of inadequately sterilized medical equipment [5]. 

Sub-Saharan Africa is a region with a high prevalence of HIV, HBV, and HCV, especially in the reproductive-age population with high fertility rates, making blood-transmitted hepatitis and HIV infection a topical issue for national and global health systems. Since the prevalence of HIV, HBV, and HCV infections are high, HCWs in these geographic regions are at higher risk for these infections [6]. Furthermore, in a systematic review conducted in Africa, the pooled 12-month prevalence estimate of occupational exposure because of percutaneous injuries ranged from 16.4% in southern Africa to as high as 67.9% in the northern Africa region [1]. It is known that HBV is the most readily transmitted virus and HIV is the least. Hence, the risk of infection in highly endemic regions from needle-stick and sharps injuries varies between 0.2 and 0.5% for HIV and increases up to 3–10% for HCV and 40% for HBV [7]. Several dozen studies have been published for certain countries, permitting analysis and summarization of reliable information on HIV, HBV, and HCV among different risk groups, including HCWs. For other countries, however, very few publications can be found, and they are frequently not only limited in terms of methodology, but also contradict each other. Studies on the prevalence of bloodborne viruses among HCWs in African countries have been limited in both the quantity and quality of the diagnostic methods used.

Another transmission mechanism, enteral, is seen with hepatitis A (HAV) and hepatitis E (HEV) viruses [8]. Transmission of hepatitis A (HA) and hepatitis E (HE) pathogens occurs in most cases from person to person through the use of contaminated drinking water or through the alimentary route. HAV is considered pathogenic only for humans, while HEV is considered to be pathogenic for both humans and animals. Thus, the fundamental difference is that HA belongs to classical anthroponoses, while HE belongs to zooanthroponoses [9]. The incidence of HA and HE correlates with poor water supply quality and unsanitary living conditions. The incidence varies from country to country and is due to socio-economic factors that affect the quality of sanitation and access to quality drinking water. In developing countries with poor sanitation and personal hygiene, the majority of children (90%) become infected with HAV before the age of 10 years [10]. Improvements in economic conditions and, consequently, sanitary conditions, usually lead to an increase in the pool of adults uninfected in childhood and lacking specific antibodies. This situation, especially in the absence of vaccination, leads to an outbreak and epidemic spread of the virus [10]. 

Hepatitis E is an equally serious global public health problem. HE outbreaks, mostly aquatic, occur in the least developed or emerging economies in Africa and Asia (endemic areas), often during the monsoon season [11]. Vulnerable population groups should be tested in a timely manner for HA and HE viral markers to prevent severe cases of the disease, possible deaths, and chronic HEV disease [12,13]. Despite the high potential of diagnostic laboratory capabilities, information on the current epidemiological situation regarding HAV and HEV in low- and middle-income countries, including countries in the African continent, is extremely limited [9,14].

The Republic of Guinea is a country located on the Atlantic coast of West Africa with a population of more than 13.6 million people [15]. Currently, Guinea remains one of the least developed countries in the world, having an economic performance characterized by a significant slowdown in growth. Like many other countries in sub-Saharan Africa, with few exceptions, only 4.1% of gross domestic product (GDP) is spent on healthcare. At the same time, in terms of GDP, the country occupies 121st place. In terms of per capita income, it occupies 174th place in the ranking of countries globally, alongside ranking 150th in terms of health spending [16]. 

Eradication of the Ebola virus disease (EVD) epidemic that raged in Guinea in 2014–2016 required unplanned material investments from the country’s health authorities. For the World Health Organization (WHO) and other organizations involved in the fight against the epidemic, the priority tasks were providing material assistance to strengthen national health systems and sending specialists, equipment, medicines, and personal protective equipment to the countries affected by the epidemic. Hospitals for patient treatment were actively deployed, and laboratories focused on express methods for detection of dangerous infectious pathogens (necessary for issuing quick results and carrying out anti-epidemic measures) were equipped [17]. However, despite the assistance provided by the global community to affected countries, setbacks were seen when assessing the contribution of the healthcare and social work sector to the country’s GDP. A significant decrease was shown from +22.7% in 2013 to a failure of −13.3%—in 2015 (most likely caused by the EVD epidemic). In 2019, the contribution was 3.2% [18]. 

The SARS-CoV-2 pandemic in 2020 was another blow to the healthcare system and workers in the Republic of Guinea [19]. During the Ebola epidemic and the SARS-CoV-2 pandemic, HCWs faced many challenges, including increased workload and personal risk. Many HCWs were infected, and some of them died [20]. The currently observed lack of adequately trained professionals means that HCWs frequently find themselves working in isolation, without peer support, while having to work longer hours. As mentioned, this reduces staff attentiveness and increases the likelihood of errors, including those that increase infection risk.

The Republic of Guinea is a region with a high prevalence of many viral infectious diseases, including those caused by HIV and hepatotropic viruses [21,22,23]. The overall risk to HCWs there from bloodborne viral infections is largely unknown. For healthcare workers in the Republic of Guinea, there are no published data regarding the HIV and viral hepatitis epidemiological situation, such as vaccination and serological status. Therefore, the aim of this work was to analyze the prevalence of HIV and viral hepatitis markers among healthcare workers in the Republic of Guinea.

## 2. Materials and Methods

The study was approved by the Ethics Committee of the Saint Petersburg Pasteur Institute and National Ethics Committee, Ministry of Health of the Republic of Guinea. The study material was 74 blood plasma samples collected from healthcare workers of the Republic of Guinea taking a refresher course on the topic “Socially Significant Infections: Viral Parenteral Hepatitis and HIV Infection”. All those examined denied anamnesis of HIV, HBV, or HCV infection.

ELISA analysis for HIV, HBV, HCV, HAV, and HEV markers involved anti-HIV, HBsAg, HBeAg, anti-HBs IgG, anti-HBcore IgG, anti-HCV total (IgM+IgG), anti-HAV IgM/IgG, and anti-HEV IgM/IgG qualitative determination, as described earlier [24,25]. For HIV RNA, HBV DNA, and HCV RNA detection, nucleic acids were extracted from blood plasma using the AmpliPrime Ribo-Prep commercial kit (CRIE, Moscow). A viral presence test was executed by real-time polymerase chain reaction (PCR) with hybridization fluorescence detection [24]. Analysis was carried out using the “Amplisens HCV/HBV/HIV-FL”, “Amplisens HIV-Monitor-FRT”, “Amplisens HAV-FL”, and “Amplisens HEV-FL” test systems (CRIE, Moscow). When viruses were detected, sequencing of the HIV polymerase (pol) gene region [26], fragments of three HCV regions (NS3, NS5A, NS5B) [27], and/or complete HBV genomes was performed [28].

Statistical data processing was carried out using the MS Excel and Prizm 5.0 (GraphPad Software, Inc., San Diego, CA, USA) software packages. The “exact” Clopper–Pearson interval was used to estimate statistical uncertainty. Results are represented as a median (Me) indicating 95% confidence interval (95% CI). The Fisher exact test or Yates-corrected chi-squared test was used to evaluate the statistical significance of numeric data obtained during paired comparison depending on sample characteristics. A probability value of *p* < 0.05 was taken as the statistical significance threshold.

## 3. Results

**Figure 1:**
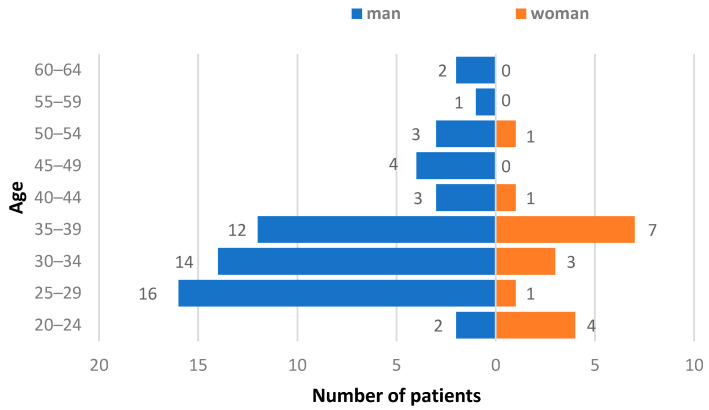
Gender and age structure of overall group.

The ages of the examined individuals ranged from 21 to 61 years; the median age was 34 years (Figure 1). The number of men in the group exceeded the number of women at 75.68% (95% CI: 64.31–84.9%) and 24.32% (95% CI: 15.1–35.69%), respectively. The mean age of women was 32.8 years. For men, it was 35.1 years. When evaluating serological markers of bloodborne infection, HIV, HCV, and HBV marker prevalence among HCWs was determined to be 4.05% (95% CI: 0.84–11.39%), 17.57% (95% CI: 9.7–28.17%), and 72.97% (95% CI: 61.39–82.64%), respectively. However, HBsAg was detected in only 6.76% (95% CI: 2.23–15.07%) of individuals. The results of HBV marker distribution analysis in the examined group are shown in Table 1.

When evaluating enteral viral hepatitis serological marker prevalence among HCWs, HAV IgG, HEV IgG, and HEV IgM were determined to be 86.49% (95% CI: 76.55–93.33%), 2.7% (95% CI: 0.33–9.42%), and 1.35% (95% CI: 0.03–7.3%), respectively. In the present study, HAV IgM-positive cases were not found.

In assessing molecular biomarker prevalence among HCWs, we saw the following: no detections of HAV RNA; 1 person for both HEV RNA and HCV RNA, accounting for 1.35% (95% CI: 0.03–7.3%) of cases; 3 people with HIV RNA (viral loads ranging from 900 to 1700 copies/mL), accounting for 4.05% of cases (95% CI: 0.84–11.39%); and HBV DNA detection in 7 people, accounting for 9.46% of cases (95% CI: 3.89–18.52%). It should be noted that in three cases (4.05%, 95% CI: 0.84–11.39%), HBV DNA was detected in HBsAg-negative individuals, while the viral load of HBV DNA was less than 15 IU/mL. It is also interesting that two of these three cases were in people co-infected with HIV + HBV.

When analyzing the nucleotide sequences of the detected viruses, it was shown that HIV was represented by the A1 subtype (two cases) and the circulating recombinant form CRF02_AG (one case); HCV was genotype 2; and the E genotype was determined for all HBV cases.

## 4. Discussion

In the Republic of Guinea, the prevalence of people living with HIV is estimated to be 1.6% [29,30]. The detection of HIV markers in 4.05% of HCWs indicates a higher risk of infection in this group. It is possible that our sample size is not sufficient to draw a definitive conclusion on the prevalence of HIV infection among HCWs, but the data obtained are sufficient to recommend a more careful attitude among healthcare workers due to an increased risk of parenteral infection. The identified HIV subtypes are consistent with data on genetic variants circulating in West Africa [21]. 

Regarding the occurrence of blood-transmitted viral hepatitis in the region, it is necessary to mention the high prevalence of hepatocellular carcinoma (HCC) in African countries, which plays a significant role in the structure of mortality associated with liver disease. Gambia is considered the most affected country, followed by the Republic of Guinea, Liberia, and Sierra Leone [31]. In countries on other continents, it is associated mainly with HCV. In African countries, HBV is a much more common cause of HCC [32,33].

Detection of HCV RNA in only one case (1.32%) is extremely low compared to previous data obtained during assessment of viral biomarker prevalence in the population (average 5.48%) [34]. However, it generally corresponds to calculated estimates of HCV prevalence: 0.72% in Southern Africa; 3.00% in East Africa; 4.14% in West Africa; and 7.82% in Central Africa [35]. The lowest documented HCV prevalence of 1.78% was in blood donors, successively rising in pregnant women (2.51%), HIV-positive individuals (3.57%), the general population (5.41%), and various high-risk groups (>10%) [36,37]. According to our research, HCV prevalence in pregnant Guinean women was 0.32% [38]. Among conditionally healthy people, it was 2.2% [39]. In general, the low prevalence of HCV is characteristic for this geographic region [40]. In this light, the low HCV prevalence in HCWs shown in this study seems natural. 

Of greatest interest is the high prevalence of HBV markers among HCWs. The main laboratory marker for the diagnosis of viral hepatitis B is the determination of HBsAg, the occurrence of which in the population varies depending on geographical region. HBsAg is a valid and predominantly used marker for HBV detection. Its detection in the blood is considered a sign of viral activity. In peripheral blood, HBsAg can be detected 2–4 weeks before the onset of clinical signs. Its concentration in acute hepatitis B (AHB) reaches maximum values and then decreases to levels undetectable by commercial test systems upon the onset of convalescence or HBsAg clearance (on average, within 4–6 months). 

However, it must be kept in mind that an absence of detectable HBsAg levels in peripheral blood does not mean complete recovery, as it may also indicate the development of occult hepatitis B infection (OBI) [41]. OBI is characterized by an undetectable HBsAg level in blood plasma in the presence of HBV DNA in liver tissue and an extremely low viral load in the blood (possibly undetectable), regardless of the presence or absence of other serological markers [42]. In this light, the problem of OBI seems to be particularly significant since HBV DNA is found in more than 75% of HBsAg-negative HCC patients [43]. What makes it particularly significant is the fact that the majority of HCC patients in the region die within a few weeks of diagnosis; i.e., mortality from HCC is comparable to morbidity. This is due to early infection with HBV, late detection of the virus, late access to doctors, and improper treatment. Some of these factors are associated with, among other things, the insufficient diagnostic methods used in the region.

Among HCWs, markers of HBV infection were detected in 72.97% of cases. At the same time, most of them (60.81%) featured anti-HBs IgG or anti-HBcore IgG antibodies, or a combination of both. Antibodies to HBsAg and their quantitative determination in the blood are used as a marker of past HBV infection or as evidence of vaccination against the virus. Antibodies to HBcAg are an indirect marker of a patient’s contact with HBV when other markers are negative [44]. The individuals we examined reported not being vaccinated against HBV. Thus, the high occurrence of these HBV serological markers in the group indicates most had contact with the virus, confirming data on pathogen prevalence in the African region. 

**Figure 2:**
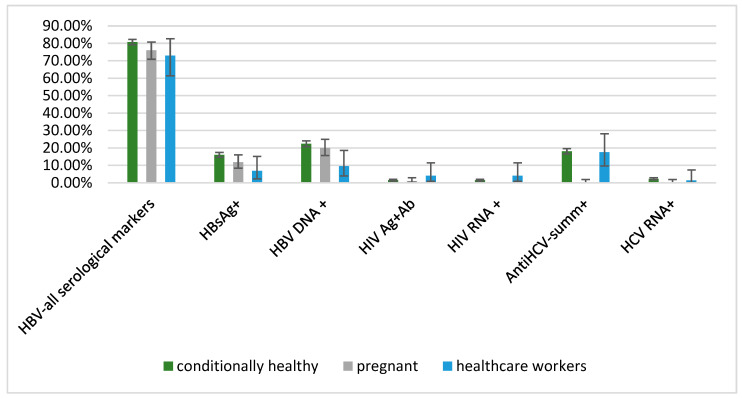
Comparative analysis of marker prevalence in blood-transmitted infections in different groups of the Republic of Guinea [28,38].

At the same time, HBsAg prevalence in this study (6.76%) was slightly lower compared to the data of other researchers. At the end of the 20th century, HBsAg prevalence in different Guinean regions averaged 16.7% [45]. Currently, the level of HBsAg occurrence in the region remains stably high. An assessment of HBsAg prevalence in various groups showed that the prevalence among HIV-infected people, diabetics, prisoners, and blood donors was 8%, 8–9%, 27.7%, and 15%, respectively [46,47]. According to data previously published by our group, HBsAg prevalence was 11.8% among pregnant women [38]; 16.4% among blood donors [48]; and 16.01% among conditionally healthy individuals [39]. Interestingly, in some African countries, HBsAg+ prevalence among infants (16.3%) and blood donors (23.4%) is significantly higher than in the rest of the population (13.6%), as described in Nigeria [49] (Figure 2).

The prevalence of HBsAg in African countries has previously been shown to be higher in men than in women, especially in rural areas. This has been associated with differences in tribal and sexual behavior between men and women [50,51]. We did not find differences in HBV marker occurrence depending on gender. It can be assumed that the lack of significant differences may be due to the high level of education and a comparative universalization of the behavior of people working in healthcare. Apparently, the social characteristics of the surveyed group did not play a significant enough role to reduce or increase viral prevalence in one sex or another. 

The distribution of HBV marker serological profiles in HCWs determined here indicates chronic hepatitis B (CHB) occurrence at a level of 5.4% (two men, two women). In order to identify the true prevalence of chronic infection, however, it is also important to take into account the occurrence of the occult form of HBV detected by molecular biological methods.

Occult hepatitis B infection prevalence in our work was also lower (4.05%) than in other risk groups. Thus, in the Republic of Guinea, data on OBI were obtained for HIV-infected patients, indicating a prevalence of up to 45.16% in this group [52]. This indicator was also determined for pregnant women; the prevalence of HBsAg-negative HBV among them was 9.84% [38]. The OBI prevalence among blood donors in the Republic of Guinea was 15.6% [48]. The relatively low HBV prevalence in healthcare workers may be due to several reasons. 

Firstly, the relatively low prevalence of HBV markers that we identified may be associated with the small size of the study group. Secondly, the majority of those surveyed were HCWs in the infectious disease field. Professional awareness may lead to increased caution and, consequently, a decreased average prevalence of infection in the group. However, the high prevalence of anti-HBs IgG or anti-HBcore IgG contradicts this assumption. Thirdly, the lack of sensitivity of the methods we used could have affected the results. The sensitivity of our HBV detection method was approximately 5 IU/mL. As such, samples with lower viral loads were left out of our view. Another reason may be the high mortality of health workers in infectious disease specialties/settings during recent epidemics; the presence of CHB, chronic hepatitis C (CHC), and HIV potentially increase the risk of death of individuals infected with Ebola virus or SARS-CoV-2.

Among the HCWs with detected HBV DNA, all had low VL (<15 IU/mL). Together with DNA, our serological findings were: three cases with HBsAg + anti-Hbcore; one case with HBsAg alone; and three cases with anti-Hbcore alone. The results obtained undeniably indicate that detection of IgG antibodies alone does not prove a past disease. Such cases require additional studies by PCR.

Anti-HAV IgG antibodies were detected among health workers in 86.49% of cases in the absence of IgM antibodies, which largely reflects the quality of the country’s hygiene standards and socio-economic development. A global analysis of sanitation and drinking water showed that hygiene and sanitation levels are still poor in sub-Saharan Africa. The majority of the population, lacking sufficient access to sources of clean drinking water, uses water from surface sources. Nearly half of Africa’s population lacks access to modern sanitation facilities or has inadequate hygiene skills [53]. These factors promote the spread of enteral viral hepatitis A and E [53].

To date, the number of published works on the prevalence of HA in West African countries is small. Most of the publications date back to the late 1970s, when the first studies were conducted to determine the prevalence of antibodies to HAV in sub-Saharan regions. Thus, despite a thirty-year difference between studies, and some improvement in the socio-economic level of the region, anti-HAV IgG prevalence is still high.

According to many researchers, while more than 80% of the population in endemic regions have antibodies to HAV, antibodies to HEV are much less common [54]. The low prevalence of IgG antibodies to HEV (2.7%) and IgM antibodies to HEV (1.35%) seen, along with the lack of data on registered waterborne HEV outbreaks in the country, are not typical for HEV waterborne transmission [55,56]. The results of the study may indicate a sporadic HE incidence in this area, possibly due to a foodborne transmission route in which the sources of infection are farm animals, in particular pigs [57,58,59]. The low HEV seroprevalence in this case may be associated with food preferences, since 85% of the population does not eat pork due to religious beliefs [60].

A significant problem remains the low awareness of health workers in African countries of viral infection transmission, including HIV and viral hepatitis, as well as knowledge about post-exposure prophylaxis in case of accidents [61]. Among the published data, there are extremely rare works devoted to the prevalence of bloodborne infection markers among HCWs in the Republic of Guinea. Along these lines, we compared our results with estimates of HIV and viral hepatitis marker prevalence among HCWs in countries in the same African region as the Republic of Guinea. In Liberia, the prevalence of CHB and CHC among HCWs was approximately 5% and 0%, respectively [62]. Among healthcare workers in Sierra Leone, 10% were positive for HBsAg. [63]. It should be noted that, as in our study, the prevalence of CHB among healthcare workers in Sierra Leone was lower than that among blood donors, pregnant women, and school children [64]. 

Similarities and differences in biomarker (HCV, HBV, HIV) detection among HCWs, and a lack of relevant data for countries in the region, may be due to: geographic differences; differences in screening and sampling methods; cultural and behavioral differences regarding risk factors; or viral transmission factors dictated by socio-cultural practices and environmental factors. It should be noted that, according to WHO estimates, only 0.3% and 6% of people infected with HBV or HCV in African countries know their serostatus, respectively [65].

## 5. Conclusions

As noted, infection and knowledge about status are problems in the regional population. More specifically, however, the higher prevalence of HIV markers in healthcare workers than in the country as a whole indicates that they are at increased risk for contracting bloodborne infections. Data on the prevalence of HIV and viral hepatitis among HCWs in the Republic of Guinea and neighboring countries are limited. Our work was a pilot study designed to fill this gap in knowledge, while showing the need to organize programs for regular screening for HIV and viral hepatitis in the population in general and healthcare workers in particular. The results obtained can serve as input data for further planning of a nationwide assessment of infection prevalence. More rigorous and extensive epidemiological studies of infection prevalence among healthcare workers are needed. 

The Republic of Guinea is among the countries facing serious shortages in human resources for healthcare. The severe healthcare worker shortage in many parts of the world is among the barriers that need to be addressed to improve primary health services. Healthcare organizations should work on strategies to increase knowledge among HCWs. Since no anti-HCV or anti-HIV vaccine is currently available to counteract viral transmission, healthcare workers should protect themselves by meticulously applying all universal prophylactic measures whenever facing potential exposure.

The limitations of the research and conclusions presented in this paper were caused by the small sample size and short study period. However, it is important to consider that this is a start for further research.

## Figures and Tables

**Table 1:** HBV serological marker prevalence and distribution in the examined group (HBsAg, HBeAg, HBcore IgG, HBs IgG).

HBV Serological Marker Prevalence	Number in the Overall Group (n = 74), Share of the Group, 95% Confidence Interval
HBsAg+	5 (6.76%, CI: 2.23–15.07%)
HBeAg+	4 (5.41%, CI: 1.49–13.27%)
HBs IgG+	32 (43.24%, CI: 31.77–55.28%)
HBcore IgG+	49 (66.22%, CI: 54.28–76.81%)
HBV serological profile distribution	
HBsAg+, HBcore IgG+	4 (5.41%, CI: 1.49–13.27%)
HBsAg+	1 (1.35%, CI: 0.03–7.3%)
HBeAg+, HBcore IgG+, HBs IgG+	4 (5.41%, CI: 1.49–13.27%)
HBcore IgG+, HBs IgG+	24 (32.43%, CI: 22.00–44.32%)
HBcore IgG+ isolated	17 (22.97%, CI: 13.99–34.21%)
HBs IgG+ isolated	4 (5.41%, CI: 1.49–13.27%)
Seronegative	20 (27.03%, CI: 17.36–38.61%)

## Data Availability

Data available on request from the authors.
